# Development of one dimensional geomechanical model for a tight gas reservoir

**DOI:** 10.1038/s41598-021-00860-z

**Published:** 2021-11-02

**Authors:** Abhiram Kumar Verma, Debasis Deb, Akshay Chandan Dey, Subrata Roy, Ajay Kumar Singh, V. L. N. Avadhani, Rajiv Ranjan Tiwari

**Affiliations:** 1 Department of Mining Engineering, IIT Kharagpur, Kharagpur, West Bengal India; 2 Centre for Excellence in Well Logging Technology (ONGC), Baroda, Gujrat India

**Keywords:** Geophysics, Civil engineering

## Abstract

Estimating rock-mechanical, petrophysical properties and pre-production stress state is essential for effective reservoir planning, development, and optimal exploitation. This paper attempts to construct a comprehensive one-dimensional mechanical earth model (1D MEM) of the Mandapeta gas reservoir of Krishna Godavari (KG) basin, India. The methodology comprises a detailed stepwise process from processing and analysis of raw log data, calibration of log-derived dynamic properties with static ones using regression models developed from tested core samples, and final rock mechanical property estimation. Pore pressure profiles have been estimated and calibrated with the Repeat formation tester (RFT) data for every thirty-five wells. Overburden and horizontal stresses have also been evaluated and calibrated using data from the Leak-off Tests (LOT) or Extended Leak-off Tests (XLOT). A menu-driven program is developed using PYTHON code for visualization and on-time revision of 1D MEM. The resulting comprehensive 1D MEM predicts and establishes the rock-mechanical properties, pore pressure, and in-situ stress values of the basin. Besides its use in planning future wells, development of the field, and yielding insight into the various well challenges, it can also be used to develop a 3D MEM of the reservoir.

## Introduction

One dimensional Mechanical Earth Model (1D MEM) is a numerical representation of the state of in-situ stress and rock physical–mechanical properties for a specific stratigraphic section in the field or basin^1,2^. These models are constructed along the wellbores based on log data^3^ to investigate the mechanical effect of rocks in wellbores such as breakouts, mud loss, sand production, wellbore stability, prediction of mud weight window for drilling, hydrofracturing initiation pressure, and well trajectories^1,3–7^. Wellbore instability can cause problems such as stuck pipes and lost circulation^8,9^.

The tectonic stress field strongly affects the stability, planning and design of wells. However, stress magnitudes and orientations are frequently not homogeneous on a reservoir scale. Still, they are modified by the presence of faults and lithological changes and contrasts in rock mechanical properties^10,11^. Plumb et al.^1^ mentioned that building MEM during the well-planning phase and revising it, in-real time has proven to be extremely valuable in delivering complex wells safely while minimizing unplanned well construction costs and accelerating learning about the field. In basic form, the MEM consists of the depth profile of the elastic and elastic–plastic rock strength parameters, pore pressure, and in-situ stresses with reference to the local stratigraphic section.

The current field under study, being a tight gas reservoir, has issues like cataclasis, clay smearing, and uncertainty about fault block connectivity, resulting in reduced production from wells. Even after extensive hydrofracturing, the wells were not able to sustain the tubing head pressure. So, the major challenge lies in efficient and economic fracturing for optimal exploitation of the hydrocarbon reserves. Proper engineering of fracture orientation and penetration requires precise estimation of in-situ stress magnitude and direction along with rock's pore pressure and mechanical properties. These input data can be used for hydrofracturing simulation before being implemented in the field. Thus, developing 1D MEM is essential for well planning and trajectory determination and addressing well-stability issues, thus reducing associated non-productive time (NPT) for modeling fracture propagation/ containment. A developed 1D MEM adds the knowledge of the field's prevailing in-situ stress and pore pressure regimes.

## Geological setting

### Krishna–Godavari (KG) basin

KG basin is a proven petroliferous structure located in the central part of the eastern continental margin of India^12^. It covers an area of 28,000 sq. km on land and 24,000 sq. km offshore up to 200 m isobaths^13^. The basin is characterized by an extensive deltaic plain formed by two east coast rivers, Krishna and Godavari, with a horst and graben structure trending NE–SW, mimicking the rifting of Indian craton from Gondwana land in early Mesozoic. The gross tectonic structure is broadly categorized as intractratonic and pericratonic^14^.

The sedimentary sequence of the KG basin ranges from Permo-Carboniferous to Recent, with sediment deposits above reaching 6 km on the coastal side and reducing towards on land. The basin consists of sediments deposited through various periods of geologic history, starting from rifting, syn-rift, drift to the late-drift stage. Sedimentation began in the Late Carboniferous to Early Permian over the Archean crystalline basement, named Kummugudem/Barakar formations. Mandapeta sandstones, the current studied area, unconformably overlie the Kummugudem and forms the reservoir rock. The Jurassic red claystone, Red bed, overlie the Mandapeta. A red bed consisting of impermeable claystone forms the cap rock. The above three formations form the nonmarine Lower Gondwana rock^12^.

**Figure 1:**
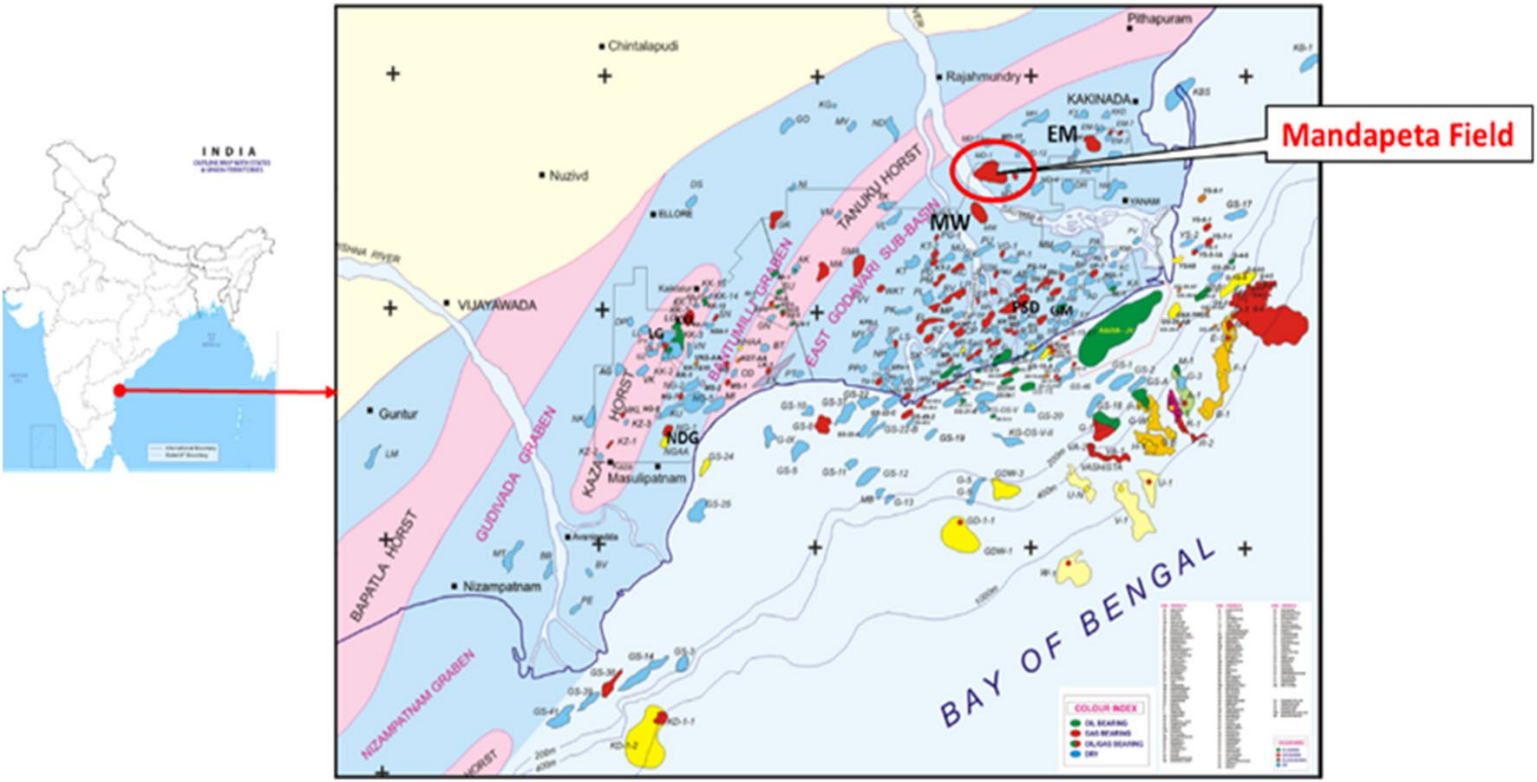
Map showing the location of the KG basin in the Indian sub-continent.

Figure 1 shows the whole basin is subdivided into three sub-basins by a series of horst/ ridges: East Godavari, West Godavari, and Krishna graben. Mandapeta field is located in the East Godavari sub-basin.

Rifting, subsequent marine invasion, sea-level rise during the Cretaceous, and late marine regression resulted in alternate shale and sandstone sequences. In the rift/syn-rift stage in Permo-Triassic to Early Cretaceous, broadly lagoonal, fluvial to brackish water sediments are deposited with a basinal tilt in sloping north east^14^. The ensuing subsidence then accommodated the syn-rift sediments in the Late Jurassic to Early Cretaceous. A significant slope reversal happened in the drifting stage by south west tilt of the basin marked by widespread marine transgression in Cretaceous with marine shale deposition in the current Raghavapuram shale sequence^12^. Sea level decrease and marine regression during Late Cretaceous resulted in deltaic arenaceous facies deposition in Tirupati sandstone. Collision of the Indian and the Eurasian plates and Matsyapuri-Palakollu fault initiated increased sediment load and accommodation in the shallow-offshore to offshore region.

### Study area (Mandapeta field)

**Figure 2:**
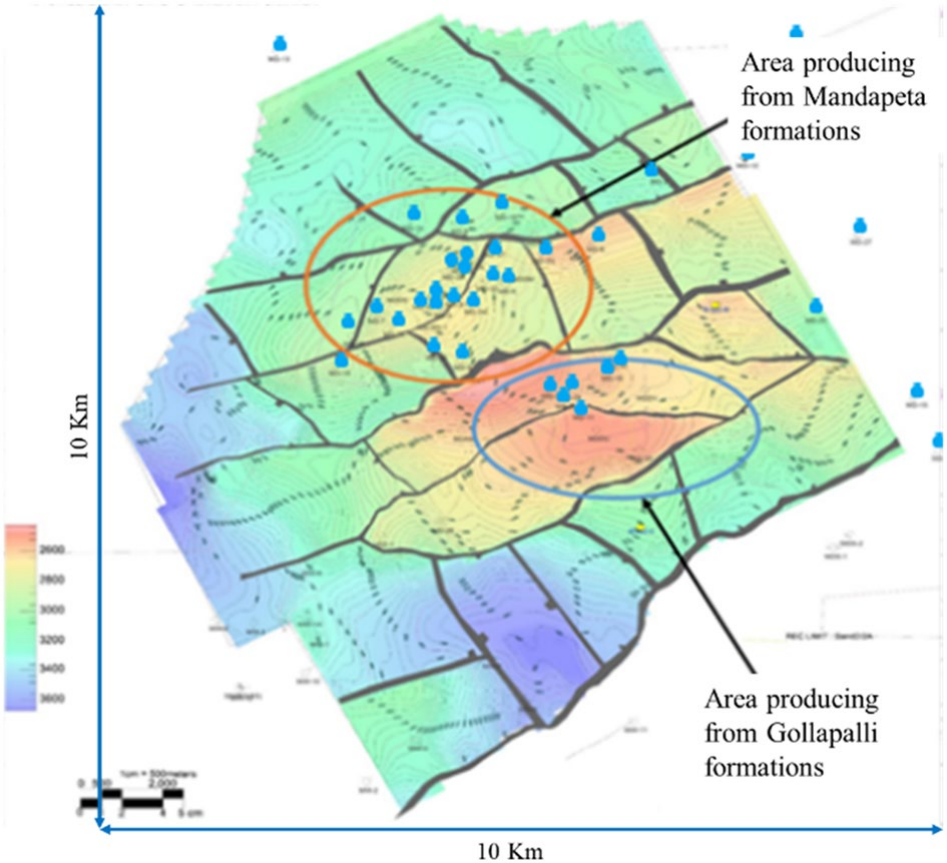
A structural map showing fault traces by black lines and well positions by cyan dots.

The studied area is located in Krishna-Godavari (KG) basin, about 20 km south–east of Rajamahendravaram (earlier Rajahmundry) town of Andhra Pradesh state of India. The aerial extent of the studied area is approximately 100 sq km having thirty-five wells, out of which twenty-two are exploratory, and thirteen are development wells. The well locations, fault traces, and fault blocks have been illustrated in the structural map on top of the Mandapeta formation shown in Fig. 2. The majority of wells are producing from the Mandapeta formations, shown in an orange circle. While the central region of the field is at a shallower depth (reddish-orange), the peripheral areas are at a higher depth (light to deep blue). The field has been subdivided into twenty-four fault blocks by the horizontal major and longitudinal minor faults.

The average thickness of the reservoir is 379 m lying between 2500 and 3500 m depth. The studied area lies in the grabenal part of East Godavari sub-basin of the KG basin, which is limited by Tanuku horst in the north-west and Draksharama horst in the east. The sediments in the graben are mainly ranging from Permo-Carboniferous to recent age. Mandapeta sandstones and Gollapalli pay are the two hydrocarbon-bearing sands developed in this prospect. The deeper low permeable principal sands-2 and 2A belong to the Permo-Triassic age, while the good quality shallow Gollapalli sands belong to the Jurassic age. Mandapeta sandstone is very coarse to medium-grained and poorly sorted. It is interbedded with claystone and shale bands. Mandapeta pay sand is divided into five sub-layers by means of intervening shales. These sub-layers are easily correlated in wells. Sand-2 is the principal sand and is tested in all the gas-bearing wells of the Mandapeta field.

**Figure 3:**
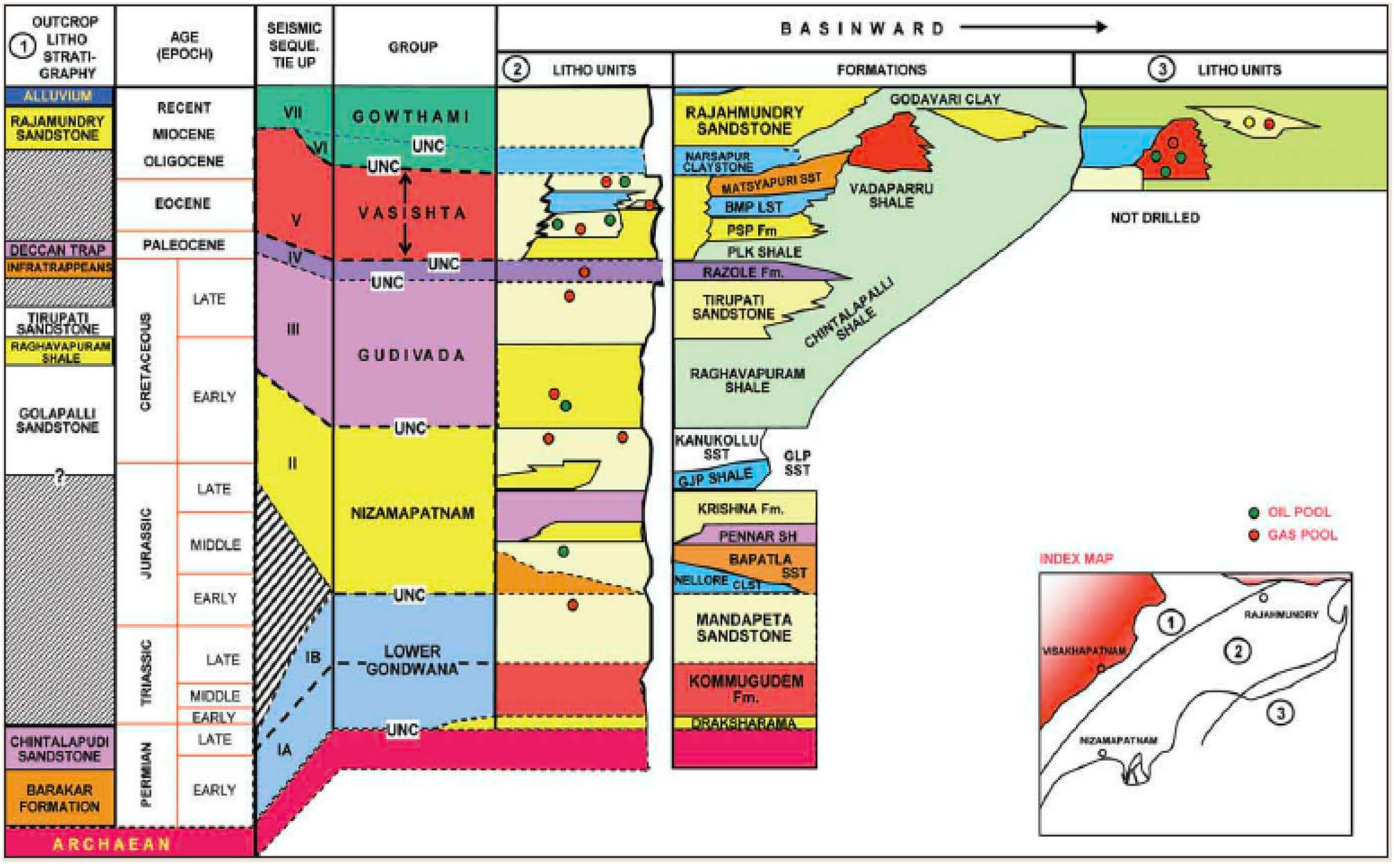
Generalized stratigraphy of KG basin from onshore to offshore^13^.

Kamaraju et al.^15^ stated that these gas sands appear to have been deposited under restricted shallow marine conditions during transgression of the sea into the north east plunging rift grabens (half grabens), forming a restricted and shallow water environment. The overall lithology of this field consists of formations from top to bottom as Rajahmundry sandstone/ Razole formation/ Tirupati Sandstone/ Raghavpuram shale/ Gollapalli formation/ Red bed/ Mandapeta sandstone/ Kummugudam formation/ Chintalpudi formation/ Basement as shown in Fig. 3. The Kummugudem-Mandapeta, belonging to the Lower-Gondwana formation shown, form the source and reservoir rock, respectively, in the Mandapeta area.

## Methodology

The mechanical earth modeling process starts with the gathering, analysis, and refinement of the available data. In the present study, the available data from thirty-five wells of the Mandapeta field includes log, drilling, and well completion reports and core samples from the reservoir horizon. But, as in typical old wells, the log data (e.g., density (RHOB), sonic travel time (Δt), and gamma-ray (GR)) are missing in many intervals along with the depth of wells. So, at the outset, a complete data set is generated by extrapolation/interpolation in places of missing values. Then, empirical relations are used to estimate the log data's rock elastic and strength properties, also called dynamic properties. To calibrate dynamic properties with static properties, available core samples from five wells belonging to the reservoir horizon are tested in the laboratory. A regression model between static and dynamic properties is established and used for all the wells for static property estimation.

The pore pressure regime of the reservoir is calculated using the traditional and widely-used Eaton^16,17^ equations. The normal compaction trend lines are drawn using sonic, in some cases, resistivity logs versus depth. The abnormal trend in the region of interest are pointed out, and normal transition time values are calculated and used for determining the pore pressure regimes.

The vertical or overburden stress is estimated by integrating the overlying density logs representing the top lithological column. Though most of the wells are vertical, some new wells are inclined or S-shaped. Thus, the measured depth (MD) is converted to corresponding true vertical depths (TVD) while integrating for vertical stress estimation. The horizontal stresses are calculated using Blanton and Olsen equations and are calibrated using the tested data like Leak-off test (LOT), extended Leak-off Tests (XLOT), and fracture integrity tests (FIT). 1D MEMs are plotted for entire depths to visualize the variations of different parameters along with the well depth.

**Figure 4:**
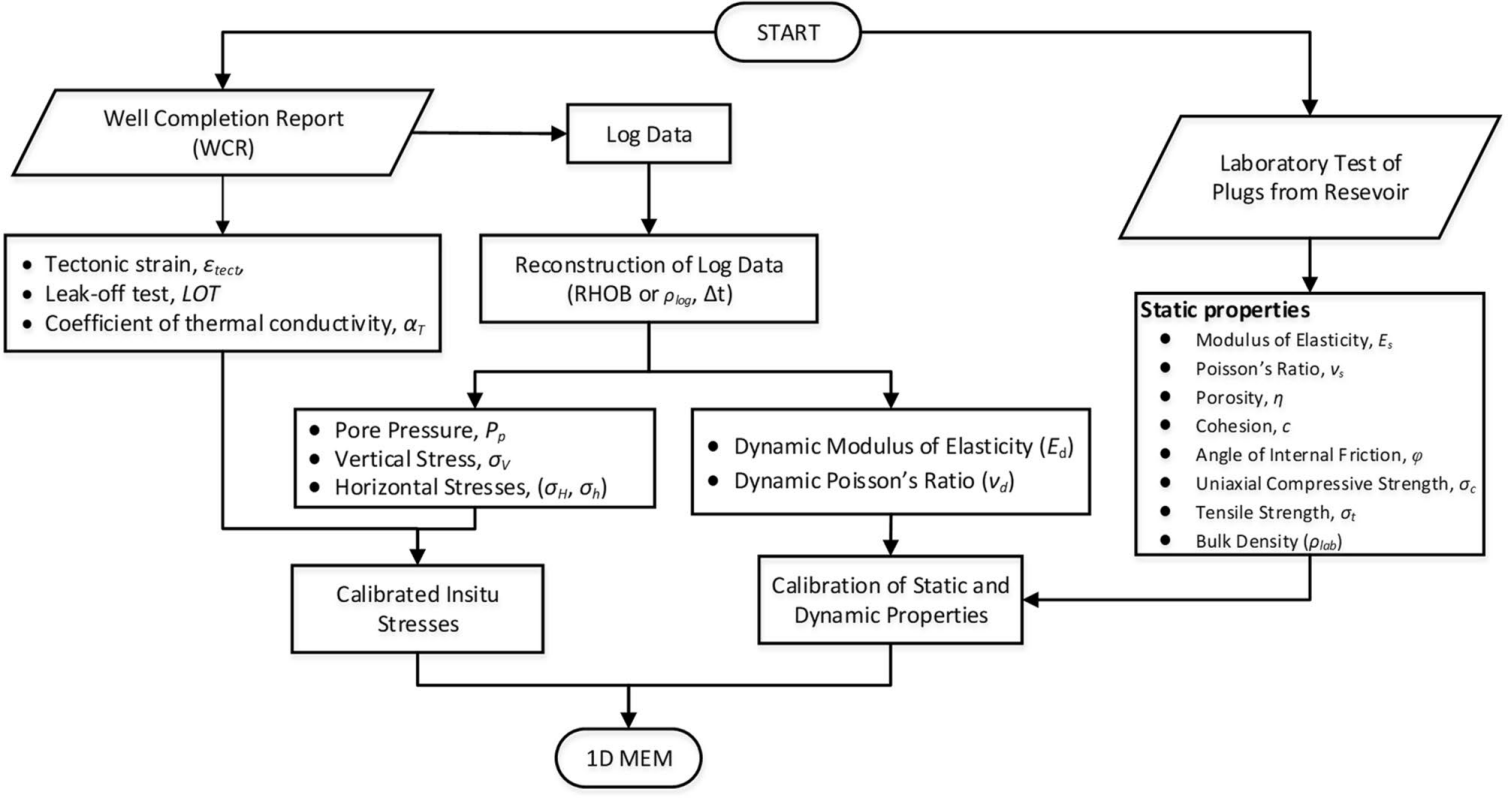
Flow chart for 1D MEM development.

A GUI-based software is also developed on the PYTHON platform, reconstructing the log data and generating 1D MEM. This GUI is also useful for further development and reconstruction of data for 3D MEM. Figure 4 shows the flow chart for the development of 1D MEM.

### Data compilation

#### Available data

The available data set from the wells are—log data, drilling and completion data reports, and core samples from the reservoir horizon. Drilling and completion reports are compiled in the form of a well-completion Report (WCR). Log data includes caliper, sonic, density, gamma-ray, resistivity, and neutron porosity. As most wells are from the late 1980s to early 1990s, there are many interspersed missing data points. In those years, the sonic tools were mostly working in P-mode. For continuous prediction of rock properties and in-situ stresses, missing data points are predicted using linear interpolation and extrapolation techniques.

#### Estimation of missing log data

The raw log data of density, sonic transition times, gamma-ray contain many missing intervals of data, often in the range of 5–20 m, with density log typically starting from 500 to 1500 m downwards. As these log data are essential for predicting rock elastic, strength properties, and stresses, each missing data must be predicted. Assuming the formation properties do not vary too much in the range of 5–20 m, a linear variation of properties has been considered, calculated by linear interpolation and extrapolation methods.

**Figure 5:**
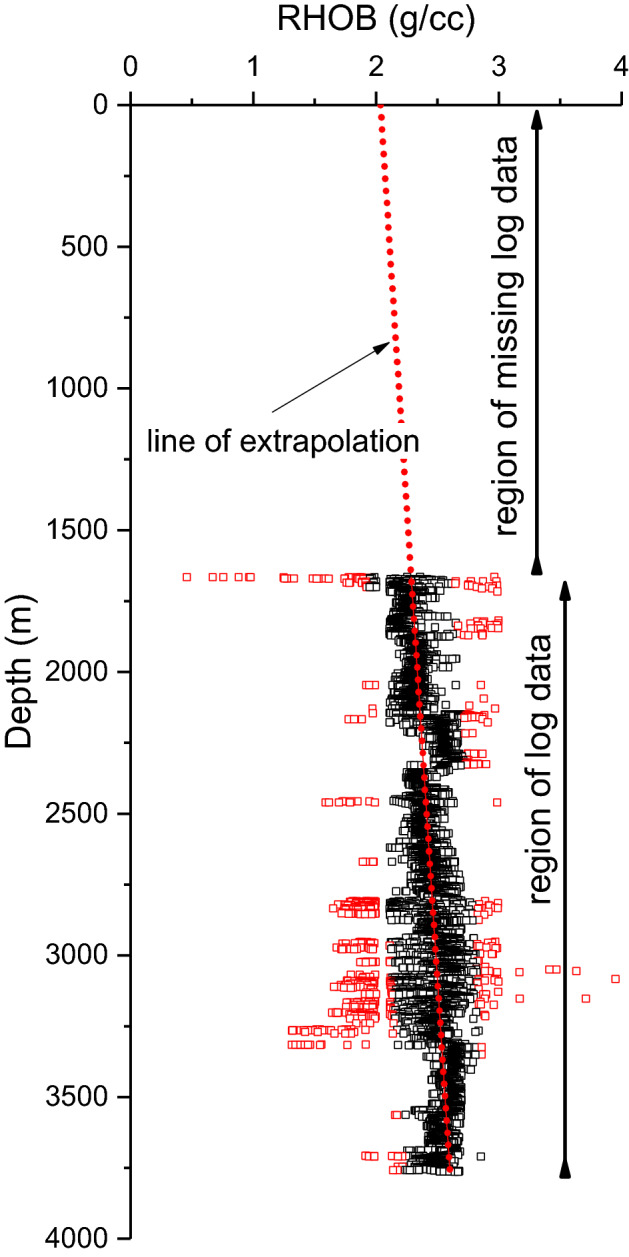
Missing RHOB data determination by extrapolation method.

To estimate vertical or overburden stress, the densities of all the overlying formations up to the surface are required. However, as the density log starts at least from 500 m downward, the missing data up to the surface has been estimated by assuming a linear variation of all overlying densities, as shown in Fig. 5. The fact that from surface up to around 1300 m are only sandstone bearing, and the absence of overpressure zones, also supports the assumptions of linear variation.

The sonic log comprises only the compressional travel time log (DTCO), as the shear log (DTSM) is absent in all but five wells. Further, the DTCO log too is missing in a couple of wells. Thus, ${V}_{p}$, which is compressional wave velocity calculated by taking the inverse of sonic transition time (${1 \mathord{\left/ {\vphantom {1 {\Delta t}}} \right. \kern-\nulldelimiterspace} {\Delta t}}$), is estimated by using empirical relationship (Eq. 1) given by Lindseth^17^ based on Gardner’s empirical data.

1 $$V_{p} = \frac{c}{{1 - \rho^{d} }}$$

where, $\rho$ is bulk density of the rock, $c,$ and $d$ are site constants depending on rock type. By iterative solution, constants $c$ is 0.296 and $d$ is − 2799.27 for one of well.

As discussed above, the shear wave transition time (DTSM) log is typically not present in old wells. However, a dipole shear sonic imager (DSI) log is present for five wells out of thirty-five. This is because, in older wells, only compressional travel time (DTCO) was recorded using sonic tools that mainly worked in P-mode. These tools worked in P and S mode only in fast formations where mud compressional velocity is less than formation shear velocity. Since this condition is very seldom achieved, the recorded velocity from these tools was generally compressional. With the introduction of dipole shear sonic tools (DSI, mark of Schlumberger), it was possible to record shear travel time (DTSM) even in slow formations. This tool directly excites flexural waves (similar to shear waves in the low-frequency range), and the mud compressional velocity criterion needs not to be fulfilled.

**Figure 6:**
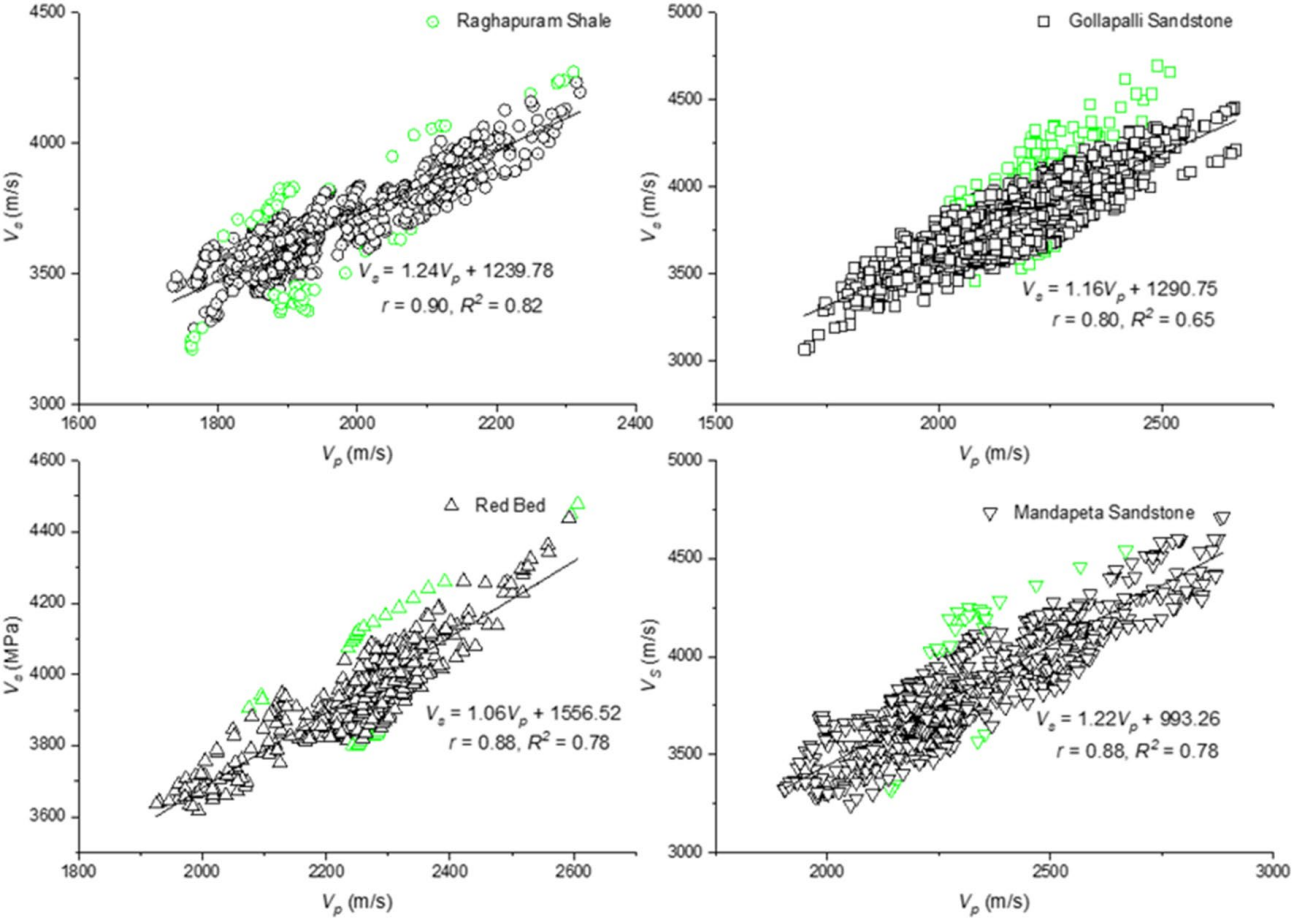
Regression model developed for ${V}_{p}$ and ${V}_{s}$ for different formations of the selected well.

Thus a regression correlation is established between compressional (${V}_{p}$) and shear wave (${V}_{s}$) velocities for each well with DSI data, and the best correlation (maximum R^2^ or determination coefficient) has been selected for estimating shear wave velocity in all the wells. The coefficient of determination for the five wells varies between 0.65 and 0.82. Again, regression relation for each formation of the selected well has been established to use these correlations for different formations, with their individual rock properties, as shown in Fig. 6. Data points marked in green are the outliers.

Lee^18^ mentioned that velocity ratio had been used for many purposes, such as a lithology indicator, determining the degree of consolidation, identifying pore fluid, predicting velocities. Usually, it depends on porosity, degree of consolidation, clay content, differential pressure, pore geometry, and other factors. The velocity ratio for dry rock or gas-saturated rock is almost constant irrespective of porosity and differential pressure. In contrast, the velocity ratio of wet rock depends significantly on porosity and differential pressure. Pickett^19^ cross plot shows that ${V}_{p}/{V}_{s}$ for sandstone is about 1.6 in low-porosity rocks, drifting to 1.8 in relatively higher porosity rocks. Gardner and Harris^20^ showed that ${V}_{p}/{V}_{s}>2$ are characteristic of water-saturated unconsolidated rocks, and values less than 2.0 indicates either well-consolidated rock or the presence of gas in unconsolidated sands. In this study, it is found that the ratio ${V}_{p}/{V}_{s}$ is 1.75 for major formations in the KG basin and each formation like Raghavpuram shale, Gollapalli sandstone, Red bed, and Mandapeta sandstone, the ratio is 1.88, 1.80, 1.82, and 1.64, respectively. Based on Pickett^19^ and Gardner and Harris^20^ works, Mandapeta sandstone having well consolidated low porosity rock and the presence of unconsolidated sands are found during drilling and coring operation.

### Determination of static rock properties and correlation with dynamic properties

Rock elastic and strength properties can be directly derived from the log data, called dynamic properties. However, as the dynamic properties are typically higher in magnitude than the actual value, they have to be calibrated to the corresponding static properties. The static properties are measured by laboratory measurements of rock cores extracted from the wells, which may not be available in all cases due to core extraction's expensive, time-consuming nature and labor-intensive operation. In such cases, lithology-based or field-specific empirical correlations are used to estimate the static rock properties^21,22^.

In the present case study, cores from five wells are present. So, instead of using empirical models from literature and calibrating the predicted properties with the laboratory-tested values, a direct regression correlation between static and dynamic properties corresponding to the particular depth of core extraction has been established. The resulting regression model has been used for all the wells to predict the static properties.

#### Dynamic elastic properties of rocks

Kolsky^23^ mentioned that a material's deformational characteristics depend on the velocity of propagation of elastic waves in a material. As given below, the logging methods^12^ determine the dynamic elastic properties based on the theory of stress wave propagation in an infinite elastic medium.

2 $$E_{d} = \rho_{d} V_{s}^{2} \frac{{3\left( {V_{p} /V_{s} } \right)^{2} - 4}}{{\left( {V_{p} /V_{s} } \right)^{2} - 1}}$$

3 $$\mu_{d} = \frac{{\left( {V_{p} /V_{s} } \right)^{2} - 2}}{{\left( {V_{p} /V_{s} } \right)^{2} - 1}}$$

where, $E_{d}$ and $\mu_{d }$ are dynamic Young’s modulus and Poisson’s ratio; $V_{p}$ and $V_{s}$ are compressional and shear wave velocities; ${\rho }_{d}$ is density of the rock. From the log data, the average dynamic properties ${E}_{d}$ and ${\mu }_{d}$ are determined for reservoir rock to be 34.68 MPa, and 0.23 respectively.

#### Static properties by laboratory tests

**Figure 7:**
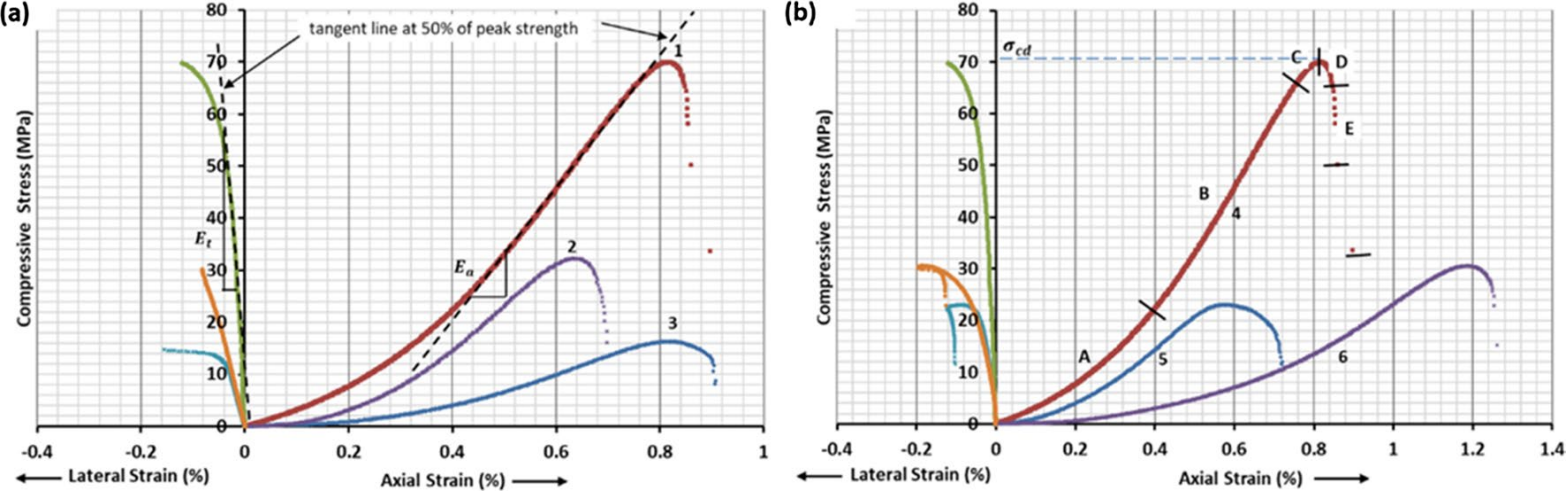
Stress vs. strain curve developed after UCS test.

In this study, static properties such as uniaxial compressive strength, tensile strength, modulus of elasticity, Poisson’s ratio, and bulk density are determined using ISRM standards^24^. Plugs of 100 mm diameter from reservoir rock are collected from five wells, and they are re-cored of 38 mm diameter plugs. Testing is performed for estimation of static properties using 3500kN servo-controlled testing machine with data logging at 100 Hz. Modern servo-controlled testing systems are used to conduct various tests in rock mechanics laboratories to find uniaxial and tensile strength. This system's choice is because the choice of control variables between a force (or pressure) and a displacement (or strain) component helps develop a complete uniaxial force–displacement curve for strain-softening material such as rock. The failed sample under compression shows the different patterns of failure like crushing, shearing, and splitting. The other failure patterns in sedimentary formation are being controlled by the micro-structure, grain size, and dissemination present. Complete stress–strain curves developed from UCS tests are shown in Fig. 7 with six representative plots out of eighteen such tests. Figure 7a three UCS test plots in which maximum, minimum, and average UCS values are obtained, whereas Fig. 7b shows three plots in which maximum, minimum, and average modulus of elasticity are found.

For UCS test, specimens are right circular cylinders having a height to diameter ratio of 2.5–3.0 and diameter preferably not less than NX core size or 54 mm. As the size of the tested specimen is 38 mm, hence the compressive strength thus obtained is corrected for 50 mm diameter sample using Eq. (4) after Hoek and Brown^25^.

4 $$\sigma_{c50} = \sigma_{cd} \left( \frac{d}{50} \right)^{0.18}$$

where, $d$ is the diameter of the tested sample in mm, $\sigma_{c50}$ is UCS for 50 mm diameter sample, $\sigma_{cd}$ is the UCS for the sample diameter $d$ tested in the laboratory.

Loads on the specimen are applied monotonically, increasing with time at a constant stress rate such that failure will occur within 5–10 min of loading. Alternatively, the stress rate shall be within the limits of 0.5–1.0 MPa/s or strain rate of 1 × 10^–4^ mm/mm/s. The UCS ($\sigma_{cd}$) of the specimen is calculated by dividing the maximum load ($P$) carried by the specimen during the test by the original cross-sectional area ($A_{o}$) of the specimen as:

5 $$\sigma_{cd} = \frac{P}{{A_{o} }}$$

A linear variable displacement transducer (LVDT) measures the axial strain produced in the test specimen due to compressive loading attached with the testing machine. In contrast, the lateral strain of the specimen is measured by two strain gauges pasted in the middle of the specimen and kept diagonally opposite to each other. The lead wires from strain gauges are connected with the data acquisition system to record the lateral strain as the load is incremented until the complete failure of the specimen. Poisson’s ratio ($\mu$) is determined as the ratio of lateral strain to the axial strain using Eqs. (6) and (7)

6 $$\varepsilon_{a} = \frac{\Delta l}{{l_{o} }}$$

7 $$\varepsilon_{l} = \frac{\Delta d}{{d_{o} }}$$

where ${l}_{o}$ is measured axial length; $\Delta l$ is the change in axial length; ${d}_{o}$ is the original un-deformed diameter of the specimen, and $\Delta d$ is the change in diameter of the specimen.

Graphically, Poisson’s ratio is determined by the ratio of axial elastic modulus ${E}_{a}$ to the lateral elastic modulus, ${E}_{l}$ using Eq. (8) as shown in Fig. 7a. In this equation, the negative sign represents negative strain in lateral direction whereas positive strain in the axial direction due to compression of the specimen.

8 $$\mu = - \frac{{E_{a} }}{{E_{l} }}$$

where the lateral elastic modulus, $E_{l}$ can be calculated as the slope of the plot between axial stress and lateral strain curve in either of the three ways to find the axial Young’s modulus.

The axial Young’s modulus, ${E}_{a}$ is defined as the ratio of the axial stress change to axial strain produced, can be calculated using any one of the three methods. The most common method of tangent Young’s modulus, ${E}_{t}$, is measured at a stress level which is some fixed percentage of the ultimate strength. It is generally taken at a stress level equal to 50% of the ultimate compressive strength as shown in Fig. 7b. Other methods to measure modulus are average Young’s modulus, ${E}_{a\mathrm{v}}$, and secant Young’s modulus, ${E}_{s}$.

Wawersik and Fairhurst^26^ classified the complete stress–strain curve in eight characteristic regions, defined in terms of mechanistic stages of fracture development based on compression tests on a range of rock types. At the same time, the post-peak behaviors of the rock are divided into two classes. For class I behavior, fracture propagation is stable in the sense that work must be done on the specimen for each incremental decrease in load-carrying capacity. For class II behavior, the fracture process is unstable or self-sustaining, and hence energy must be extracted from the material to control fracture. Figure 7b shows the class I behavior in the studied plugs and shows five characteristics regions as **A** to **E**. In regions **A** and **B,** no fabric changes in rock specimen are observed by compression. As the pore spaces are closed in region **A** and maximum resistance is offered by the sample in region **B**., Fracture will be evident before the onset of region **C**. The formation of many isolated fractures marks the failure in regions **C** and **D**. The modes of fracture in all samples are macroscopic shear fractures. The information about fracture formation for these plugs is helpful to design fracture propagation. Here, the ratio of compressive strength to tensile strength for the reservoir rock is 11.

Table 1 summarises the static properties determined for the reservoir rock. As per ISRM classification^24^, UCS between 25 and 50 MPa is considered a medium-strong rock that cannot be scraped or peeled with a pocket knife. The specimen can be fractured with a single firm blow of the geological hammer.

**Table 1:** Summary of the physico-mechanical properties.

Parameters	${\sigma }_{c50}$(MPa)	${E}_{l}$(GPa)	$\mu$	${\sigma }_{t}$(MPa)	$\rho$(kg/m^3^)
Average	29.09	5.88	0.14	2.63	2255.91
Std. Div	17.07	2.92	0.10	1.97	168.73

#### Relation between static and dynamic properties

Various researchers^21,22,27–29^ have studied the regression relationship between static and dynamic properties of rock. Differences between static and dynamic properties are attributed to the liability of static measurements to be influenced by rock discontinuities and their non-linear mechanical behavior. Davarpanah et al.^27^ mentioned that correlation depends on rock type; in the case of igneous rock, the correlation is non-linear and follows power-law, whereas sedimentary rock follows non-linear logarithmic and power law. Eissa and Kazi^22^ mentioned that the dynamic modulus is generally higher than the static, but there are instances where the opposite is true. Test results from Lama and Vutukuri^29^ show that ${E}_{d}$ is greater than ${E}_{s}$ with a variation up to 300% and ${\mu }_{d}$ is also slightly greater than ${\mu }_{s}$.

**Figure 8:**
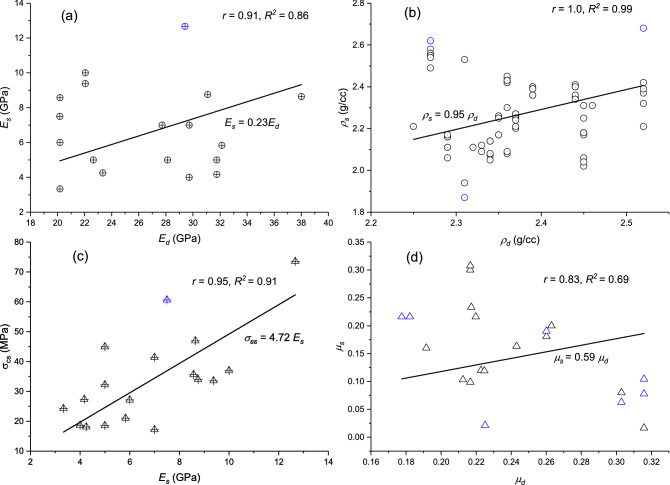
Regression model for static and dynamic properties such as (**a**) modulus of elasticity, (**b**) density, (**c**) UCS and modulus of elasticity, and (**d**) Poisson’s ratio.

Figure 8 shows the estimation of static properties from the dynamic properties determined from log data as shown by Eqs. (9–12). The outliers from the scatter plot are identified if the standardized residual is greater than $2.5\sigma$ and shown as blue data points.

9 $$E_{s} = 0.23E_{d}$$

10 $$\rho_{s} = 0.95\rho_{d}$$

11 $$\sigma_{cd} = 4.72E_{s}$$

12 $$\mu_{s} = 0.59\mu_{d}$$

This study shows that dynamic properties like modulus of elasticity, Poisson’s ratio, density are considerably greater than the static properties. On average dynamic modulus of elasticity is 3.9 times while Poisson’s ratio is 1.68 times the static properties. Dynamic density is almost the same as the static density by a factor of 1.04. The above relations (Eqs. 9–12) estimate the static properties from dynamic ones for all the wells.

### Pore pressure and normal compaction trend

Pore pressure (PP) is the fluid pressure in the pore spaces in porous formations. It is one of the most important parameters for drilling plans and geomechanical and geological analysis^30^. Zoback^10^ has enumerated that the value of pore pressure at a depth, $z$ is usually described in relation to hydrostatic (or normal) pressure, a pressure associated with a column of water from the surface to the burial depth. Hydrostatic pore pressure ${P}_{h}={\rho }_{w}gz$ increases with depth at a rate of roughly 10 MPa/km or 0.44 psi/ft, however, it’s value depends on the salinity of the water. At relatively shallow depth, pore pressure is hydrostatic, implying a continuous, interconnected column of pore water from the surface to that depth. The pore pressure increases with depth rapidly for further increase in depth, indicating that these formations are hydraulically isolated from shallower depth. With further increase in depth, pore pressure reaches a value close to the overburden stress; a condition generally referred to as hard overpressure.

The fundamental theory for pore pressure prediction is based on Biot’s and Terzaghi’s effective law^31,32^. This theory indicates that pore pressure ${P}_{p}$ in the formation is a function of total stress or overburden stress (see Eq. (2)) and the effective vertical stress, ${\sigma }_{e}$. Considering, Biot effective stress coefficient $\alpha (0<\alpha \le 1)$ pore pressure ${P}_{p}$ is shown in Eq. (13).

13 $$P_{p} = \left( {\sigma_{v} - \sigma_{e} } \right)/\alpha$$

Conventionally, $\alpha = 1$ is assumed for geo-pressured continuity. The effective vertical stress, $\sigma_{e}$ is correlated to well log data, such as resistivity, sonic travel time/velocity, bulk density, and drilling parameters (e.g., $d -$ exponent). Chilingar et al.^33^ mentioned that $d -$ exponent is a dimensionless number which depends on drilling penetration rate ($R$) in ft/h, bit diameter ($D$) in inches, the weight of bit ($W$) in lb and rotary speed ($N$) in rpm then $d -$ exponent is expressed as $d = log\left( {R/60N} \right)/log\left( {12W/10^{6} D} \right)$. Basically, it’s a measure of the ease of penetration of the drilling tool in a formation.

Different prediction methods are developed for estimating $P_{p}$, as for example, acoustic travel time/ velocity based method by Hottmann and Johnson^34^ and Gardner et al^35^ and resistivity based method by Eaton^16,36^, sonic compressional transit time by Eaton^16^, sonic interval velocity method by Bowers^37^ and sonic seismic transit time based on Miller method by Miller^38^ as well as by Tau model by Dutta^39^.

**Figure 9:**
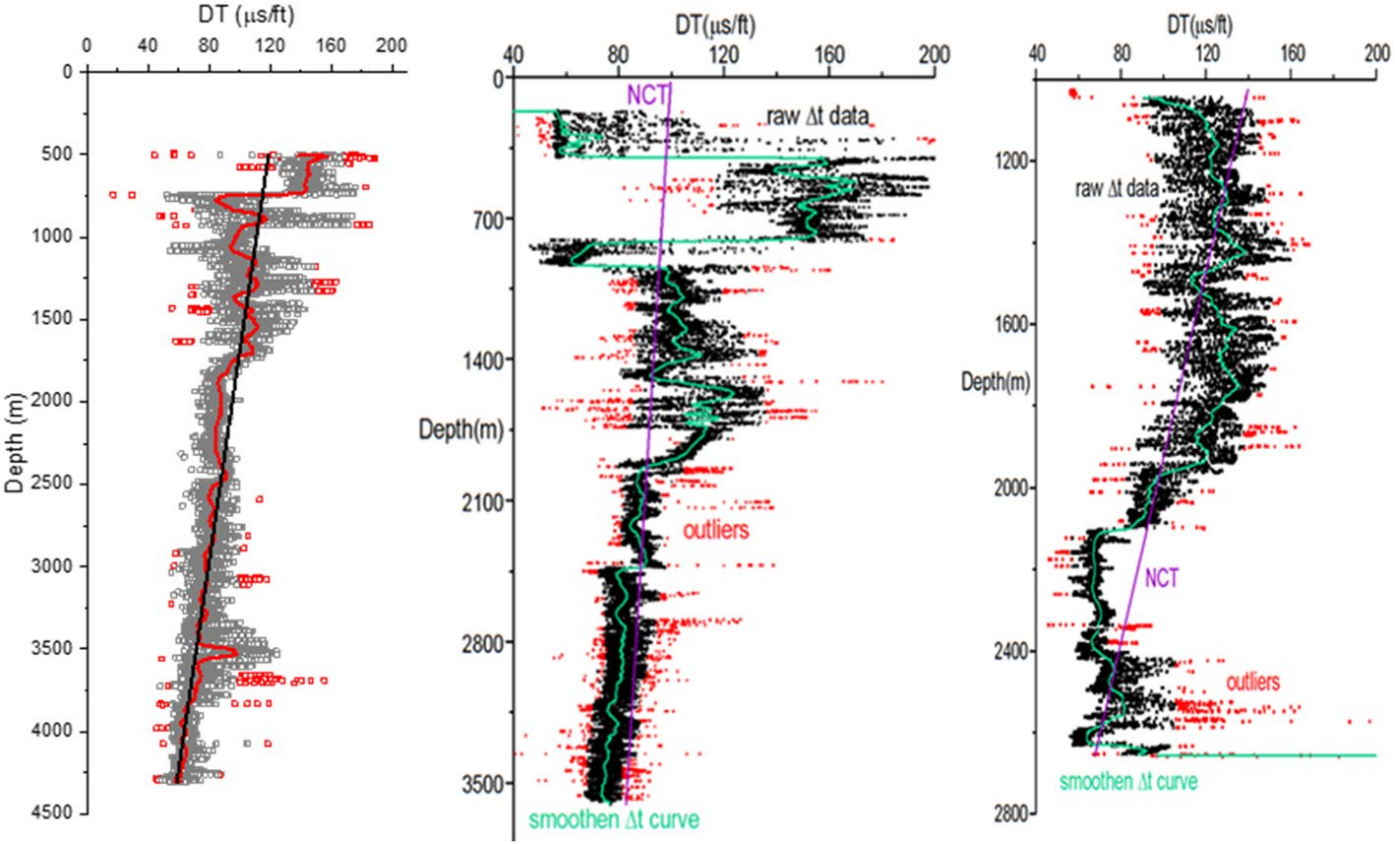
Smoothened ∆t vs. depth curve and NCT plot for well # 01, 27, and 30, respectively.

Eaton’s resistivity method^36^ is applicable for a young sedimentary basin if the normal shale resistivity is known and this method is used widely in the petroleum industry and gives a realistic estimation. The predictive equation for pore pressure based on Eaton slowness method is reproduced in Eq. (14).

14 $$P_{p} = \sigma_{V} - \left( {\sigma_{V} - P_{h} } \right)\left( {\frac{{\Delta t_{n} }}{\Delta t}} \right)^{n}$$

where, $\Delta {t}_{n}$ is compressional travel time calculated from NCT (normal transit time/normal compaction trend), and $\Delta t$ is compressional travel time obtained from a sonic log. The underlying theory is any deviation from NCT means abnormal compaction, thus the presence of abnormal pore pressure. For depth *D* (in m), the normal compressional travel (NCT) time is estimated for thirty five wellbores by linear regression. Equation 15 shows NCT for three wells having extreme intercept and slope as shown in Fig. 9.

15a $$\Delta t_{n} = 126.72 - 0.0158D$$

15b $$\Delta t_{n} = 99.82 - 0.004D$$

15c $$\Delta t_{n} = 184.87 - 0.044D$$

Leaving the top few hundred meters, the NCT plot of all thirty-five wells shows that $\Delta {t}_{n}$ decreases linearly with the increase in depth, with deviations at the hydrocarbon-bearing zones. Three representative plots of 3 wells are shown in Fig. 9, illustrating the NCT trend. Well 01 and 27 turn out to be dry, thus, showing no deviations in NCT trend below 2000 m. Whereas well 30 shows significant deviations from the NCT trend below 2000 m. The fact that this gas reservoir comprises extremely tight sandstones with permeability as low as 0.01md, also in some cases, produces a linear NCT despite hydrocarbon occurrence.

A previous study in this region by Singha and Chatterjee^40^ estimated high pore pressure gradients varying from 11.85 to 12.80 MPa/km for six east and west Godavari wells sub-basins in India. However, in this study pore pressure gradient across thirty-five wellbore is 8.42 ± 4.65 MPa/km spread over twenty-four fault blocks. The average pore pressure in the reservoir is 33.62 MPa with minimum and maximum values of 17.12 MPa and 43.61 MPa, respectively. This fluctuation in reservoir pore pressure may be due to the fault blocks being hydraulically unconnected with fault seals which act as a barrier to the fluid flow.

### Determination of In-situ stress

Three principal stresses act in the subsurface: vertical or overburden stress, maximum horizontal stress, and minimum horizontal stress. The relative magnitudes of these principal stresses control the majority of the subsurface phenomenon. A tight gas reservoir like the present field, requiring extensive hydrofracturing, in-situ stress plays a vital role in controlling fracture growth^41^. Generally, the in-situ stress is represented by the magnitude and direction of these principal stresses and changes with depth^42^. Though the magnitude and direction of in-situ stress can be taken from the World Stress Map (WSM) project database^43^, they typically only represent the regional trend of insitu stress, not variations within the field. For reference, a recent study by Singha and Chatterjee^40^ with data of three wells in the KG basin found a vertical stress gradient of 21.0 to 22.85 MPa/km. The minimum horizontal stress to vertical stress magnitude ratio varies between 64 and 76%, whereas maximum horizontal stress to vertical stress magnitude varies from 90 and 92%. The breakout derived orientation of major horizontal stress from two well varies from N14^o^E to N22.5^o^E in KG-basin. The following sections discuss the method of estimation of the three principal stresses.

#### Vertical or overburden stress

The bulk density $\rho \left(z\right)$ varies with rock type as well as depth. The total vertical stress ${\sigma }_{v}$ at any depth from the surface is calculated by integrating all the overlying densities up to the measurement point. For a deviated well, this integration has to be done along with the true vertical depth (TVD), not the measured depth, starting from the surface as shown by Eq. (16). For a nearly flat surface topography, this vertical stress represents one of the principal stress directions.^21^

16 $$\sigma_{v} = g\mathop \smallint \limits_{0}^{z} \rho \left( z \right)dz = \overline{\rho }gz$$

where, $\overline{\rho }$ is average bulk density, and $z$ is TVD from the surface.

#### Horizontal stresses

Literature study points out several empirical methods which are used to estimate the minor ${(\sigma }_{h})$ and the major ${(\sigma }_{H})$ horizontal stresses such as (1) conventional method; (2) Blanton Olson method^15,44^ (3) vertical transverse Isotropy method^45^; and (4) Harikrishnan method^46^ Maximum horizontal stress being more difficult to predict, stress polygon method are used for constraining its value.

In conventional method Eqs. (17 and 18) are obtained by solving the linear poroelastic equation with the assumption that vertical stress is equal to horizontal stress with an additional tectonic stress term to account for the tectonic component. It also implicitly assumes that the only source of horizontal stress is overburden stress and pore pressure, conspicuously ignoring the effect of thermal gradient (thus, the burial history). In addition, the added tectonic stress component, which can be expressed in terms of corresponding strain values in Eqs. (17 and 18), imply that implemented strain value is constant across all the lithologies, which is not the case given the different elastic properties of other rock formations.

17 $$\sigma_{H} = \left( {\frac{\nu }{\nu - 1}} \right)(\sigma_{v} - \alpha P_{p} ) + \alpha P_{p} + \left( {\frac{E}{{1 - \nu^{2} }}} \right)(\varepsilon_{H} + \nu \varepsilon_{h} )$$

18 $$\sigma_{h} = \left( {\frac{\nu }{\nu - 1}} \right)(\sigma_{v} - \alpha P_{p} ) + \alpha P_{p} + \left( {\frac{E}{{1 - \nu^{2} }}} \right)(\varepsilon_{h} + \nu \varepsilon_{H} )$$

To correct these inherent problems with the conventional method, a strain corrected model assigns different strain values depending on rock properties and incorporates the effect of the thermal gradient. Blanton Olsen method^44^ has been adopted in this study to estimate the horizontal stress magnitudes, as shown in Eqs. (19, 20, 21 and 22). At first, it calculates the elastic tectonic strain (${\epsilon }_{tect}$) at the point of physical stress measurement (LOT, XLOT, or minifrac), as the ${\sigma }_{h}$,$\mu$, ${c}_{1}$, and ${c}_{2}$ values are known. If the tectonic strain is compressive, i.e., ${\epsilon }_{tect}>0$, then the major and the minor horizontal stresses are expressed by Eq. (19) and (20).

19 $$\sigma_{H} = {c_{1}}{\epsilon}_{tect} + c_{2}$$

20 $$\sigma_{h} = c_{1} \mu{\epsilon}_{tect} + c_{2}$$

where, $c_{1} = E/\left( {1 - \mu^{2} } \right)$, the combined horizontal geostatic stress and thermal stresses can be expressed as $c_{2} = \left( {\mu \sigma_{v} + \left( {1 - 2\mu } \right)\alpha P_{p} + E\alpha_{T} \Delta T} \right)/\left( {1 - \mu } \right)$, $E$ and $\mu$ are the modulus of elasticity and Poisson’s ratio of rock respectively, $\alpha_{T}$ is the coefficient of thermal expansion, and its value is 5.56 × 10^–6^/^o^F for sandstone and 5 × 10^–6^/^o^F for shale, $\Delta T$ is the difference in temperature at the measuring depth with the surface temperature.

Similarly, if the tectonic strain is tensile, i.e., ${\epsilon}_{tect} < 0$, then the major and the minor horizontal stresses are expressed by Eqs. (21) and (22).

21 $$\sigma_{H} = c_{1} \mu{\epsilon}_{tect} + c_{2}$$

22 $$\sigma_{h} = {c_{1}}{\epsilon}_{tect} + c_{2}$$

After the estimation of horizontal stresses for all the thirty-five wells, it is observed that the tectonic stress field is hydrostatic, and the ratio of horizontal stresses ${\sigma }_{H}/{\sigma }_{h}$ is 1.05 ± 0.03. Hence, the tectonic (horizontal) stress field are not dominating the stress regime. Similarly, the ratio of ${\sigma }_{H}/{\sigma }_{\mathrm{v}}$ is 0.83 ± 0.08 and ${\sigma }_{h}/{\sigma }_{\mathrm{v}}$ is 0.78 ± 0.06. This indicates that, the horizontal stresses are considerably less than the vertical stress. Thus, this result is also in line with the established fact that this Mandapeta field lies in a passive margin tectonic setting and is an extensional basin. In other words, the field is characterized by a normal faulting stress regime.

## 1D MEM plots

As discussed, one dimensional mechanical earth model (1D MEM) is a graphical representation of in-situ stresses, pore pressure, and rock physical–mechanical properties along with the depth of a wellbore. In other words, it comprises plotting the variations of different relevant rock properties, pore pressure, and stresses along with the subsurface depth, sometimes just plotting different properties of interest for contrast and comparisons and drawing appropriate practical conclusions for enhanced insight into the subsurface environment.

### Machine learning for predicting missing data and GUI development

**Figure 10:**
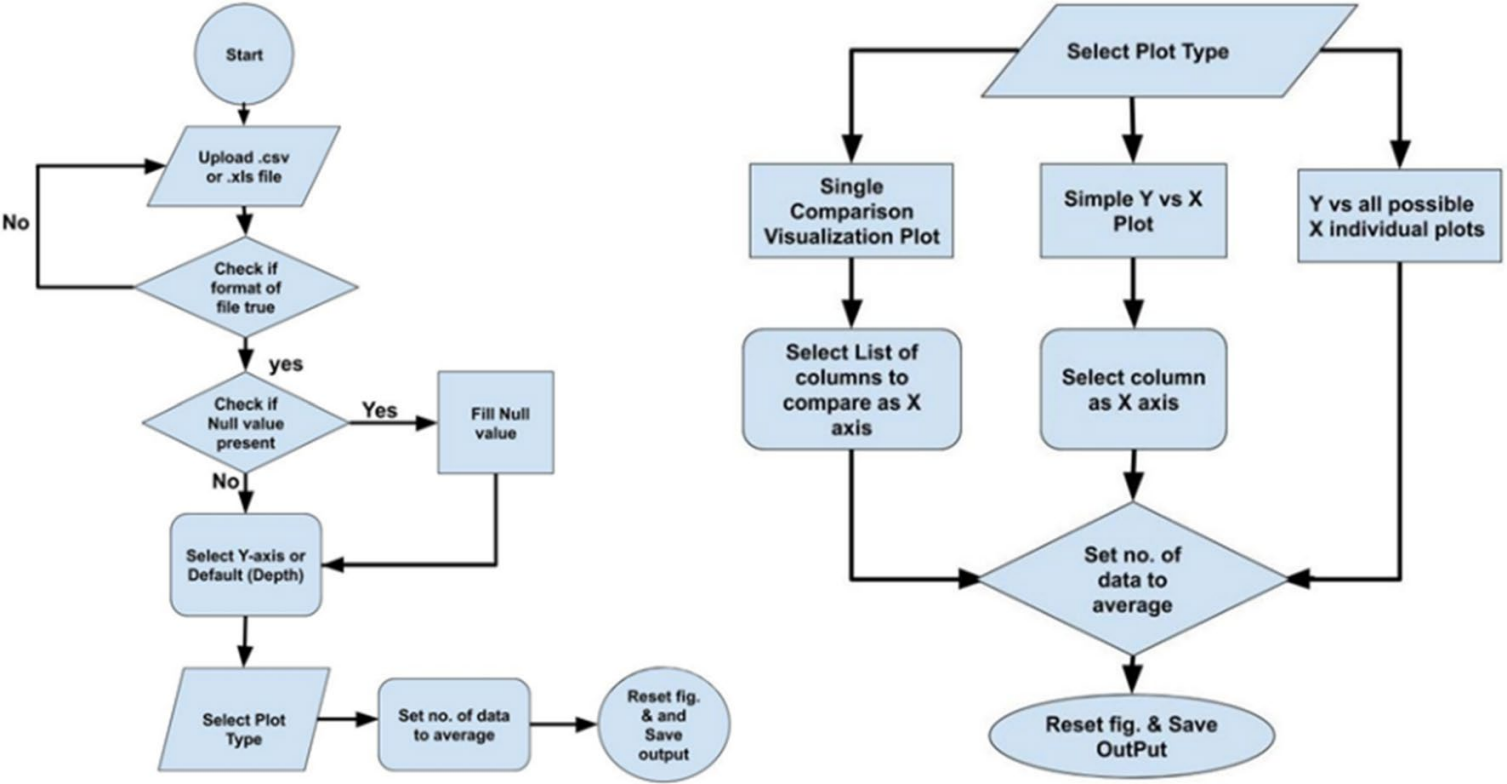
Flow chart of TKinter GUI based on PYTHON platform.

As in 1D MEM, multiple plots are plotted and compared simultaneously. To simplify the plotting process, a GUI (graphical user interface) has been prepared to customize the presentation of datasets allowing the user to select properties from the menu driven user interface. In addition, machine learning has been used to identify the missing data points and perform their descriptive statistics. The developed GUI is written on PYTHON scripts, uses Matplotlib for plotting, machine learning algorithms for training and prediction of missing data, and uses Tkinter to make a user interface through which users can instruct the machine to execute the desired program. To use this application, a user needs to upload the datasets as CSV or XLS files and perform the desired task with the help of few clicks of buttons as represented in the flow chart as shown in Fig. 10.

Missing data are predicted using machine learning methods such as KNNImputer and RandomForestImputer, and MissingValuesHandler libraries. These libraries are written in PYTHON on top of Scikit-Learn, with a Tensorflow dependency.

The KNNImputer method provides imputation for filling the missing values using the k-Nearest Neighbors approach. By default, a euclidean distance metric that supports missing values, nan_euclidean_distances, is used to find the nearest neighbors. Each missing feature is imputed using values from n_neighbors nearest neighbors that have a value for the feature. The feature of the neighbors is averaged uniformly or weighted by distance to each neighbor. If a sample has more than one missing feature, the neighbors can differ depending on the imputed feature. When the number of available neighbors is less than n_neighbors, and there are no defined distances to the training set, the training set average for that feature is used during imputation. Suppose at least one neighbor with a defined distance, the weighted or unweighted average of the remaining neighbors will be used during imputation. If a feature is always missing in training, then it is removed during transform.

The RandomForestImputer method of MissingValueHandler library has been used to automatically distinguish between different types of datasets, i.e., categorical vs. numerical, and decide whether a classifier or regressor is suited for the task. The mathematical logic for predicting missing data points is performed using (1) univariate imputation and (2) multivariate imputation. In the case of the univariate imputation method, the imputation of missing data in a variable is dependent on regression of the other data points present. This method is applicable for fewer missing points. Whereas in the case of multivariate imputation, the missing value of a variable that depends on the properties of the other variables is estimated using machine learning methods.

**Figure 11:**
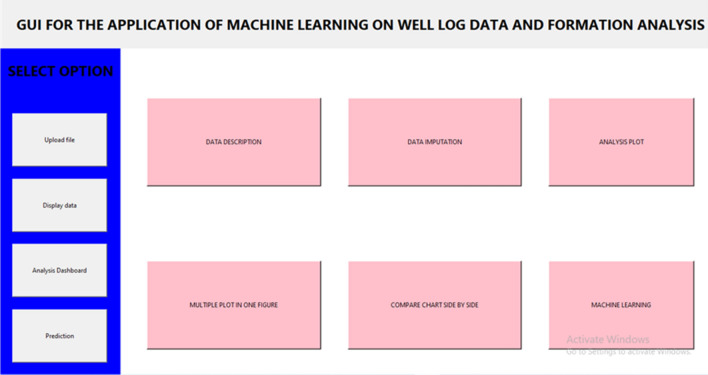
Main window of GUI.

Figure 11 shows the main window of the GUI in which tabs are designed for various operations. Various features, including the different methods for imputation for missing data and data analysis for statistical methods, are incorporated.

### Property and stress plots:

**Figure 12:**
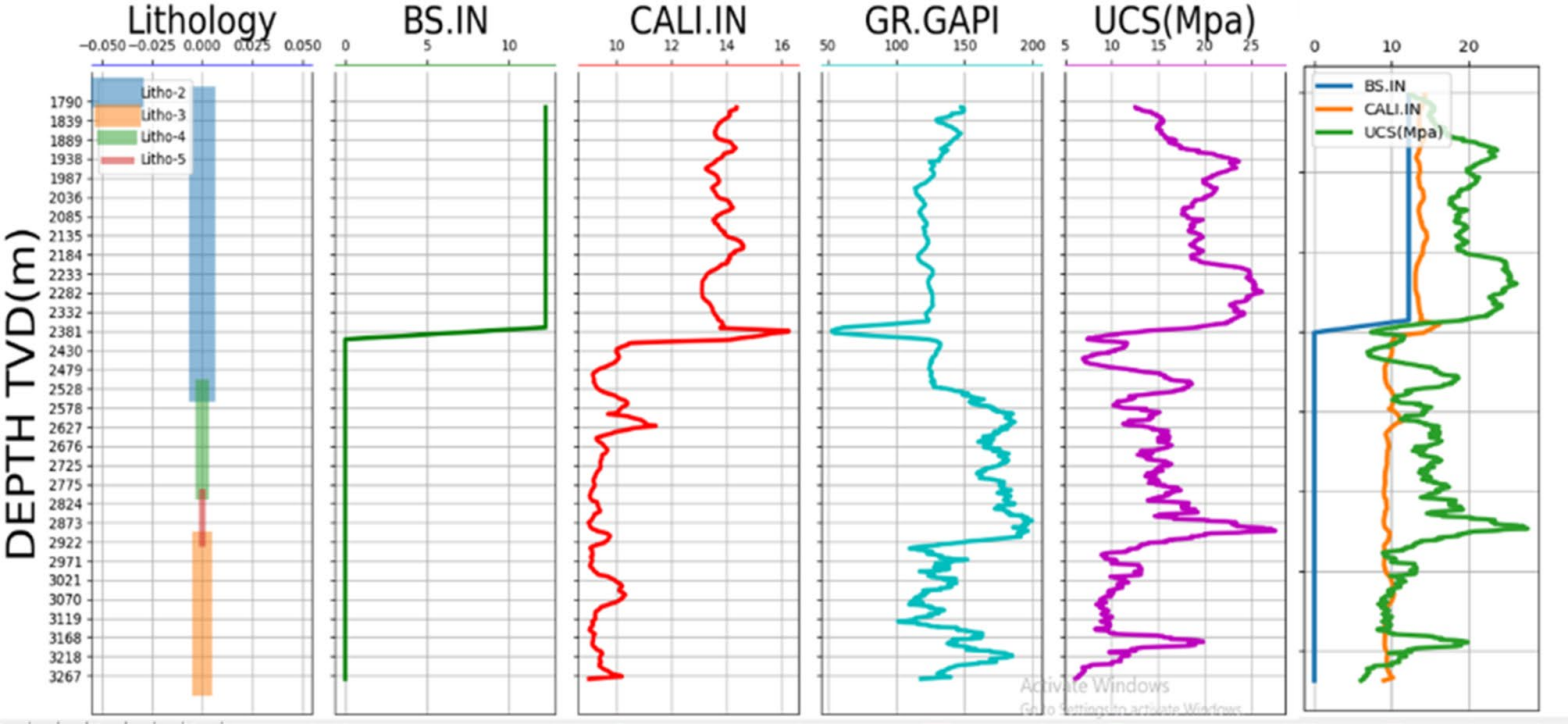
1D Mechanical earth model.

**Figure 13:**
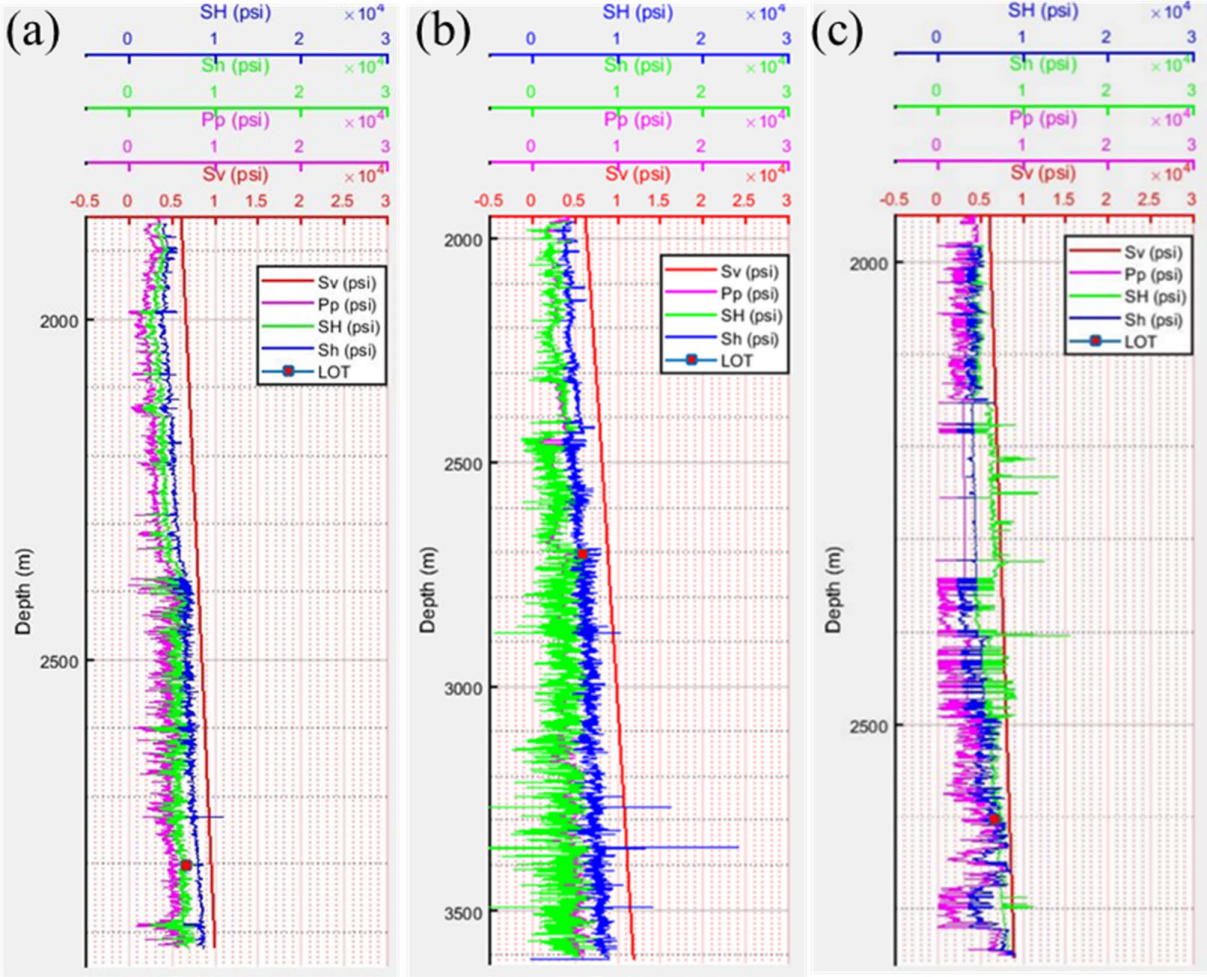
Estimation of in-situ stress and pore pressure for well (**a**) MD-01, (**b**) MD-27, and (**c**) MD-30.

After compilation of datasets, 1D MEM plots with a different variable can be selected as shown in Fig. 12 in the standard format, such as (1) single variable plot, (2) multiple variable plots with different x- and same y-axis. In Fig. 13, an example plot of stresses and pore pressure values of 3 different wells (MD-1, MD-25, and MD-30) are given. It can be seen the vertical stress is the maximum principal stress showing a linear variation marked by the straight line. The horizontal stresses follow the same trend but with different magnitudes. The pore pressure value is the minimum in value. The red dots show the measured minimum horizontal stress values, which fall in line with the predicted ones.

## Conclusions

This study presents a detailed workflow of developing a one-dimensional mechanical earth model for a reservoir. A stepwise process right from compiling a complete dataset by finding the missing values of log data, using regression model for estimating shear wave velocity, calculating dynamic properties, and establishing a regression model to predict the static properties, predicting the pore pressure profile and stresses along the wellbore profile. A customized GUI is developed using machine learning algorithms to predict missing data and developed for 1D MEM. This successfully applied workflow can also be applied in other fields with similar initial data to build a comprehensive 1D MEM.

The study shows that overburden stress is the dominant principal stress, while the horizontal stress magnitudes are approaching hydrostatic conditions. Thus, the prevailing stress regime of the field is a normal faulting regime, which is in confirmation with underlying tectonics and established view. The pore pressure profile throughout the basin seems to be nearly hydrostatic except in the reservoir formations. In some cases, pore pressure abnormality has not been observed even in the reservoir formations themselves due to the very low permeability of reservoir rock. The study establishes the nature of the basin in the Mandapeta field, which is summarised as follows:

- The average velocity ratio is 1.75 for the lower formations of the KG basin, and the average velocity ratio for Raghavpuram shale, Gollapalli sandstone, Red bed, and Mandapeta sandstone is 1.88, 1.80, 1.82, and 1.64, respectively. Based on Pickett’s (1963) and Gardner and Harris (1968) works on classification based on velocity analysis, Mandapeta sandstone has well consolidated low porosity rock and the presence of unconsolidated sands.
- The estimated average vertical stress gradient is 12.65 ± 2.53 MPa/km. Horizontal stresses are determined using the Blanton-Olson equation and calibrated with hydrofracturing test results. The horizontal stress ratio of ${\sigma }_{H}/{\sigma }_{h}$ is 1.05 around reservoir rock with a standard deviation of 0.03. Similarly, ${\sigma }_{H}/{\sigma }_{\mathrm{v}}$ and ${\sigma }_{h}/{\sigma }_{\mathrm{v}}$ are 0.83 and 0.78, respectively.
- The pore pressure gradient across thirty-five wellbores turns out to be 8.42 ± 4.65 MPa/km spread over twenty-four fault blocks. The average pore pressure in the reservoir section is 33.62 MPa with minimum and maximum values of 17.12 MPa and 43.61 MPa, respectively. The fluctuation in reservoir pore pressure may be due to probable fault seal and extensive cataclasis among these fault blocks, which acts as a barrier to the fluid flow.
- The average depth of the reservoir is 2811 m with an average density of 2255.91 kg/m^3^; uniaxial compressive strength 29.09 MPa is classified as a medium-strong rock as per ISRM; tensile strength is 2.63 MPa; modulus of elasticity is 5.88GPa; Poisson’s ratio is 0.14. The compression to shear wave velocity ratio across all the wells lies between 1.42 to 1.81, whereas Poisson’s ratio lies between 0.12 and 0.25. The compressive to the tensile strength ratio of reservoir rock is 11.
- Complete stress vs. strain plots show that the reservoir rock is classified as class I type brittle rock. It means that hydraulic pressure must be applied to the wellbore for stable fracture propagation to keep the crack open for permeability enhancement.
- The dynamic properties come out significantly greater than the static properties. On average dynamic modulus of elasticity is 3.9 times while Poisson’s ratio is 1.68 times the static value. Dynamic density is almost the same as static density, with a ratio of 1.04.
- Developed GUI is based on Python scripts, uses Matplotlib for plotting, machine learning algorithms for training and prediction of missing data, and finally uses Tkinter to make user interface through which user can instruct the machine to execute the desired results program.

The study results can be used in planning, drilling, and completing future wells and aiding in developing the field in general. The predicted in-situ stresses and existing hydrofracturing design can be used in the future, which is a significant challenge in this tight gas reservoir. The expected pore pressure and stress distribution can optimize the drilling risk and reduce the associated NPT, adding valuable knowledge about the field and the subsurface environment.
